# Unveiling Nasal Glial Heterotopia: A Pathological Perspective

**DOI:** 10.7759/cureus.59341

**Published:** 2024-04-30

**Authors:** Sanjay Deotale, Rashmi Wankhade, Pratibha Dawande, Nandkishor Bankar

**Affiliations:** 1 Pathology, Datta Meghe Medical College, Datta Meghe Institute of Higher Education and Research, Nagpur, IND; 2 Microbiology, Jawaharlal Nehru Medical College, Datta Meghe Institute of Higher Education and Research, Wardha, IND

**Keywords:** encephalocele, intranasal, nasal glioma, extranasal, nasal glial heterotopia

## Abstract

The uncommon, non-hereditary congenital abnormalities known as nasal glial heterotopias (NGH) are composed of heterotopic neuroglial tissue. Typically, NGH manifests in infancy, but occasionally it can also be seen in older children and adults. To rule out intracranial extension, magnetic resonance imaging (MRI) and computed tomography (CT) scans should be performed. Numerous cases have been documented where NGH was mistakenly identified as encephaloceles, teratomas, dermoid cysts, capillary haemangiomas, and even desmoids. A proper clinical, sonological, and even CT and MRI evaluation can lead to a near-final diagnosis; nonetheless, surgical excision and histological confirmation are the gold standards. We report a rare case of a firm, subcutaneous, non-tender, non-reducible midline 2 x 2 x 1 cm swelling with bluish-red skin near the root of the nose that was not affected by posture or pressure. Encephalocele, NGH, and dermoid were the differential diagnoses made based on the oedema found on CT and MRI scans. Histopathology provided a conclusive NGH diagnosis. The instance illustrates the significance of histology as the gold standard for NGH diagnosis.

## Introduction

Nasal gliomas, also called nasal glial heterotopias (NGH), are uncommon congenital lesions resulting from aberrant embryonic development. They typically consist of anomalous aggregates of normal glial tissue located far from the central nervous system and lacking any intracranial linkage [1]. NGH accounts for approximately 5% of all congenital nasal masses, with an incidence of congenital nasal masses estimated to be one in 20,000 to 40,000 live births [2, 3]. Reid initially reported NGH, a rare congenital disease, in 1952. Schimdt first used the term "glioma" in 1900 [4]. The nose, its surrounding area and nasopharynx are the most frequently affected areas. There are three types of nasal gliomas: intranasal (30%), extranasal (60%), and mixed (10%). The nasal dorsum is the most typical location for extranasal forms, while the lateral wall of the middle turbinate is the most common location for intranasal forms. Lips, tongue, scalp, and oropharynx are further uncommon sites where heterotopic brain matter can be found [5].

In infants, midline lesions consist of gliomas, encephaloceles, dermoid cysts, and haemangiomas [6]. The location affects the clinical presentation, which is non-specific. Nasal blockage, nasal polyps, chronic sinusitis, and chronic otitis media are common clinical symptoms [7]. Early in life, complete surgical excision is recommended to prevent complications like meningitis, brain abscess for intranasal gliomas, and aesthetic issues for extranasal gliomas. NGH is frequently misdiagnosed as haemangiomas, midline dermoid, or encephaloceles. Congenital abnormalities in the skull generate extracranial hernias of the meninges and/or brain, known as encephaloceles. Due to their intracranial connection, when they cry, strain, or squeeze the jugular vein, the mass expands and pulses. Another differential for NGH is a nasal dermoid cyst. Based on histology, the distinction can be made because NGH displays mature glial tissue and the dermoid cysts are lined by keratinized stratified squamous epithelium that contains skin tissues or dermal appendages such as hair follicles, sebaceous glands, and sweat glands [8]. A near-final diagnosis can still be made even after using a proper, systematic technique that includes sonography, computed tomography (CT), and magnetic resonance imaging (MRI). It is therefore recommended that every resected specimen be submitted to a histopathological examination and, if necessary, immunohistochemistry (IHC) to obtain 100% diagnosis accuracy. Herein we describe a case of a three-month-old male presented with NGH.

## Case presentation

**Figure 1:**
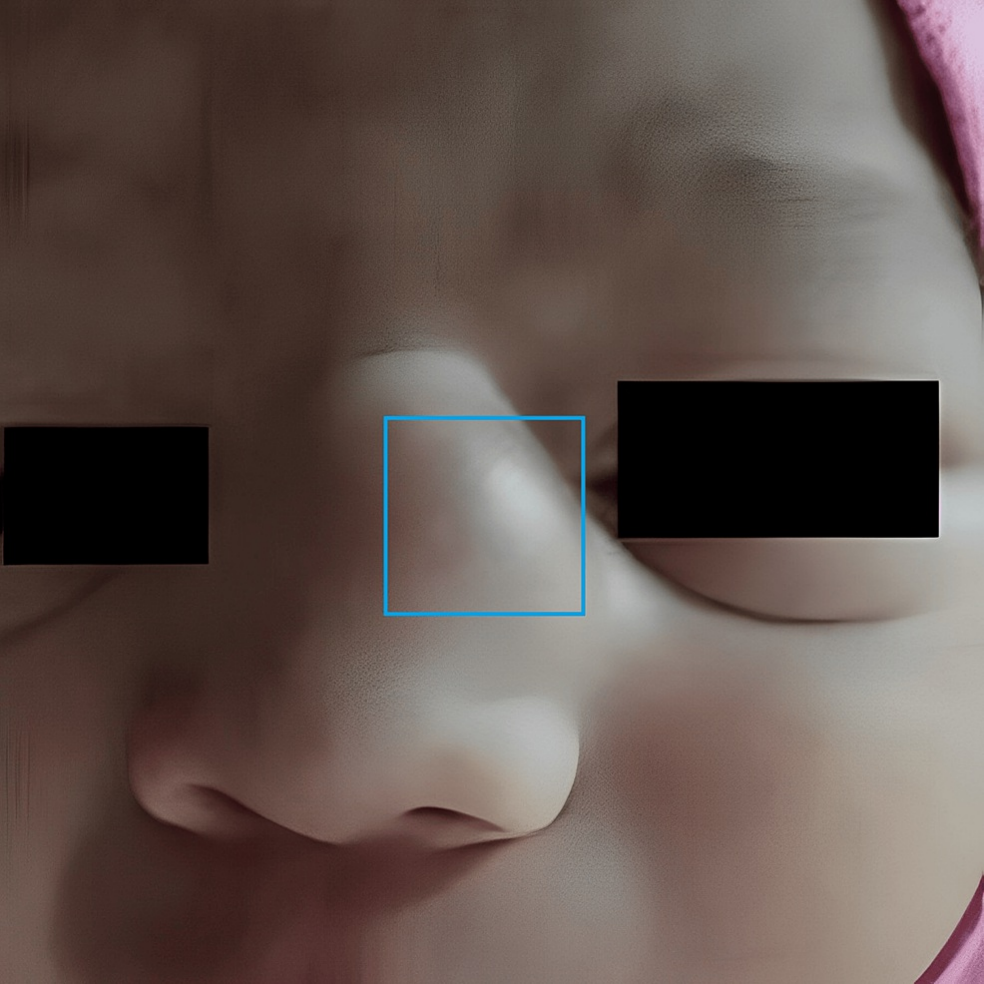
Extra nasal mass on the left side of the root of the nose (blue box)

A three-month-old boy presented with a mass localised extra nasally on the left side of the root of the nose since birth (Figure 1). There was no history of epistaxis, nasal obstruction, breathing difficulties, or feeding difficulties. The swelling did not worsen in response to crying or obstruct his vision. The swelling was non-tender, non-pulsatile, non-discharging, and firm in consistency. It was non-compressible and non-reducible in response to pressure or posture. The swelling gradually increased to the present size of 2 x 2 x 1 cm. The intercanthal distance was increased. Routine haemograms, urine analysis, and biochemistry testing were unremarkable. A 1.5 x 1.5 x 0.2 cm soft tissue tumour without cerebral or intranasal extension was revealed by a non-contrast computed tomography (NCCT) scan of the head. There were no visible bone abnormalities in the paranasal sinuses, base of the skull, or nasal cavity. Following surgical excision, the specimen was sent for histopathological analysis.

On gross examination, it was a globular soft tissue mass measuring 2 x 2 x 1 cm, non-encapsulated, with an unremarkable exterior surface. The cut section showed homogeneous greyish-white areas with no signs of necrosis, haemorrhage, or calcification.

**Figure 2:**
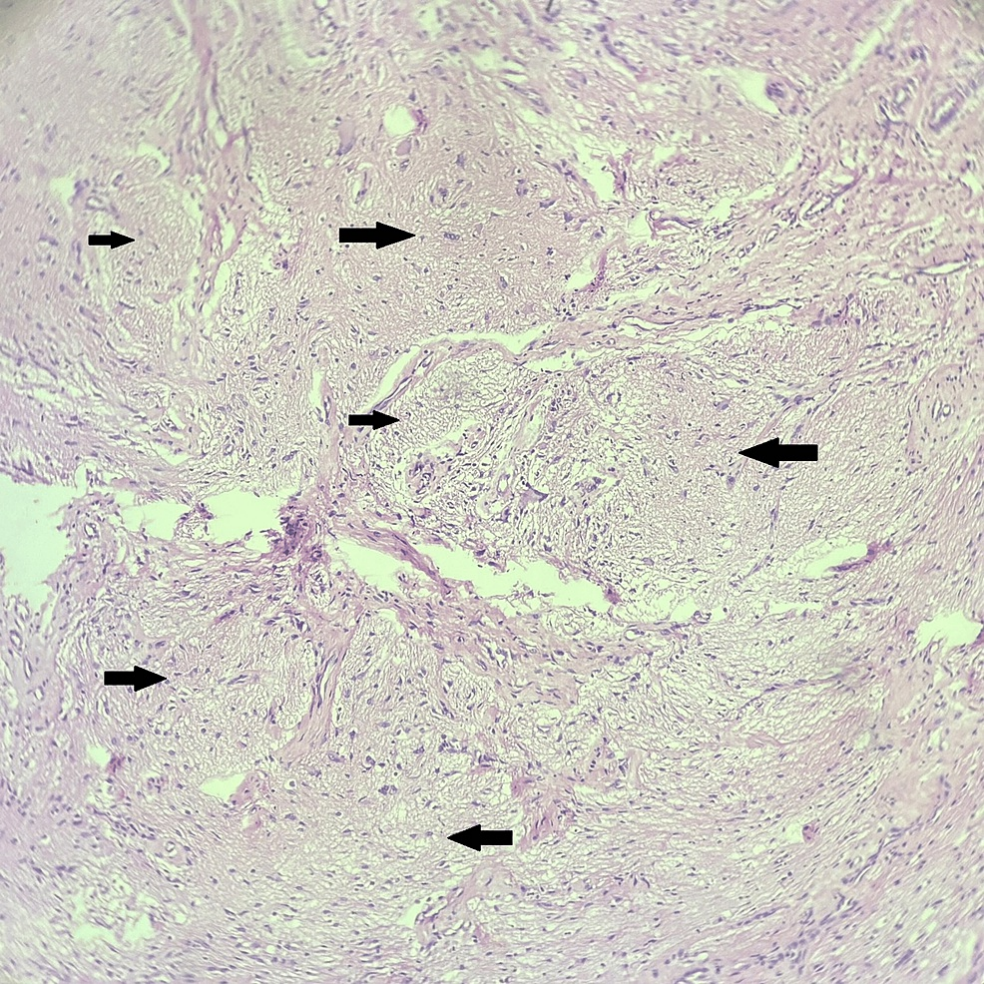
Histopathological photomicrograph showing nests and lobules of glial tissue (black arrows) (haematoxylin and eosin, 100×)

**Figure 3:**
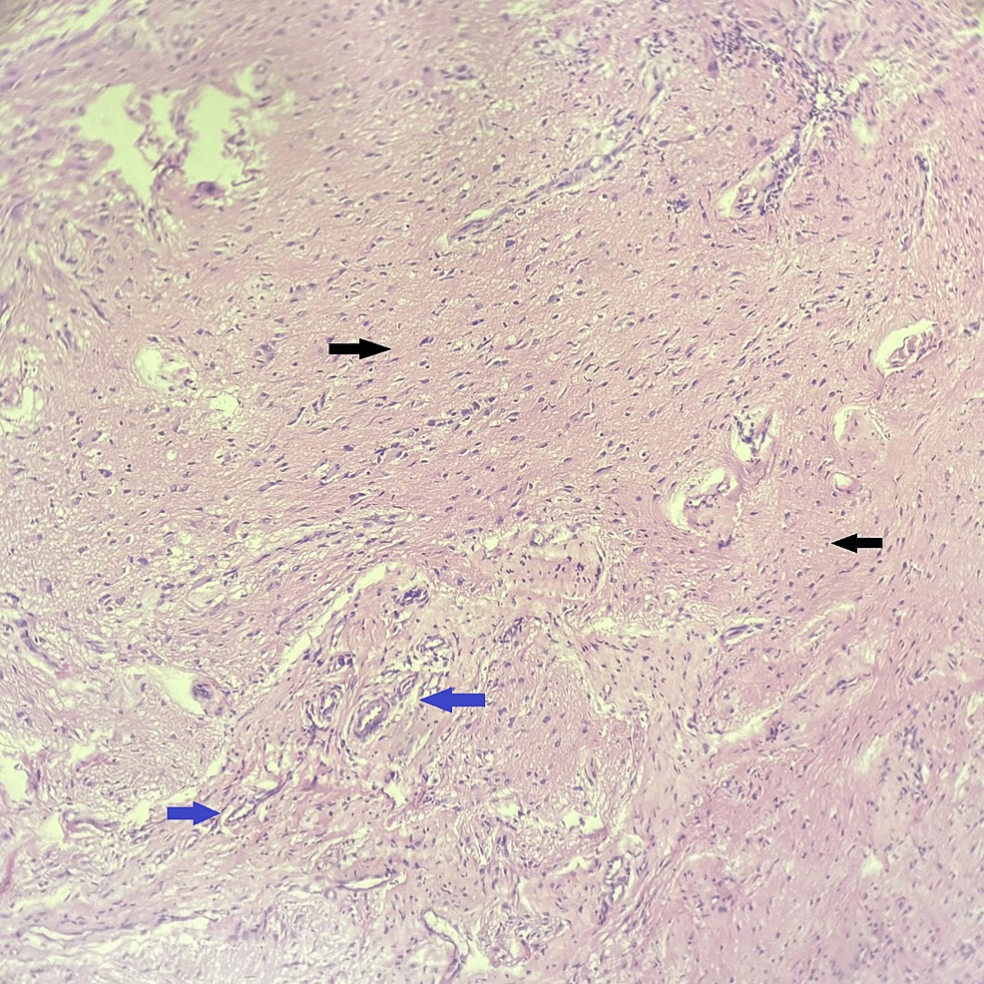
Histopathological photomicrograph showing glial tissue (black arrows) and fibrovascular stroma (blue arrows) (haematoxylin and eosin, 100×)

**Figure 4:**
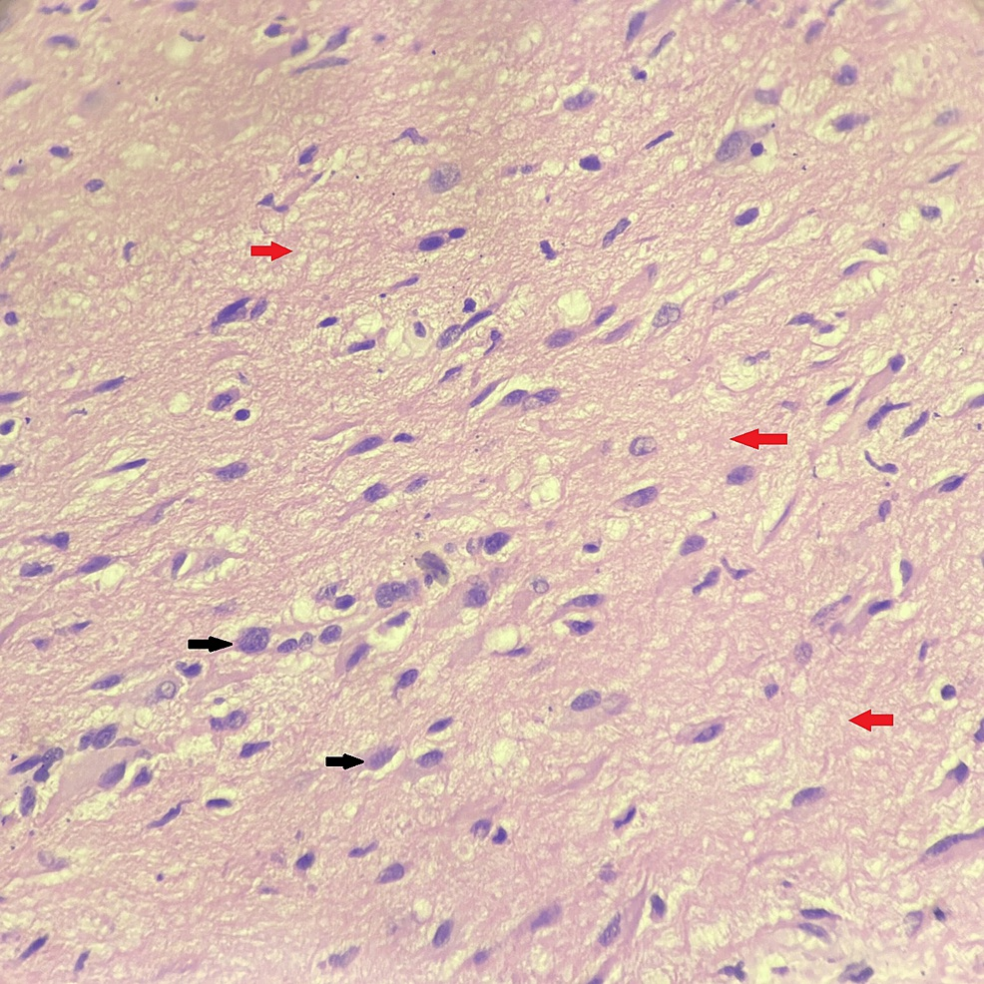
Histopathological photomicrograph showing astrocytes dispersed in a fine neurofibrillary matrix. Astrocytes show round to oval nuclei with the variegated appearance of cytoplasm (haematoxylin and eosin, 400×) Black arrows showing astrocytes dispersed in a fine neurofibrillary matrix. Red arrows showing neurofibrillary matrix.

Microscopic examination of the sections stained with haematoxylin and eosin (H&E) revealed glial tissue with astrocytes on a fibrillary background and fibroconnective tissue. There were no calcification, fibrosis, or inflammatory cells. There were no necrotic areas or mitotic figures. The final diagnosis of NGH was made (Figures 2-4).

**Figure 5:**
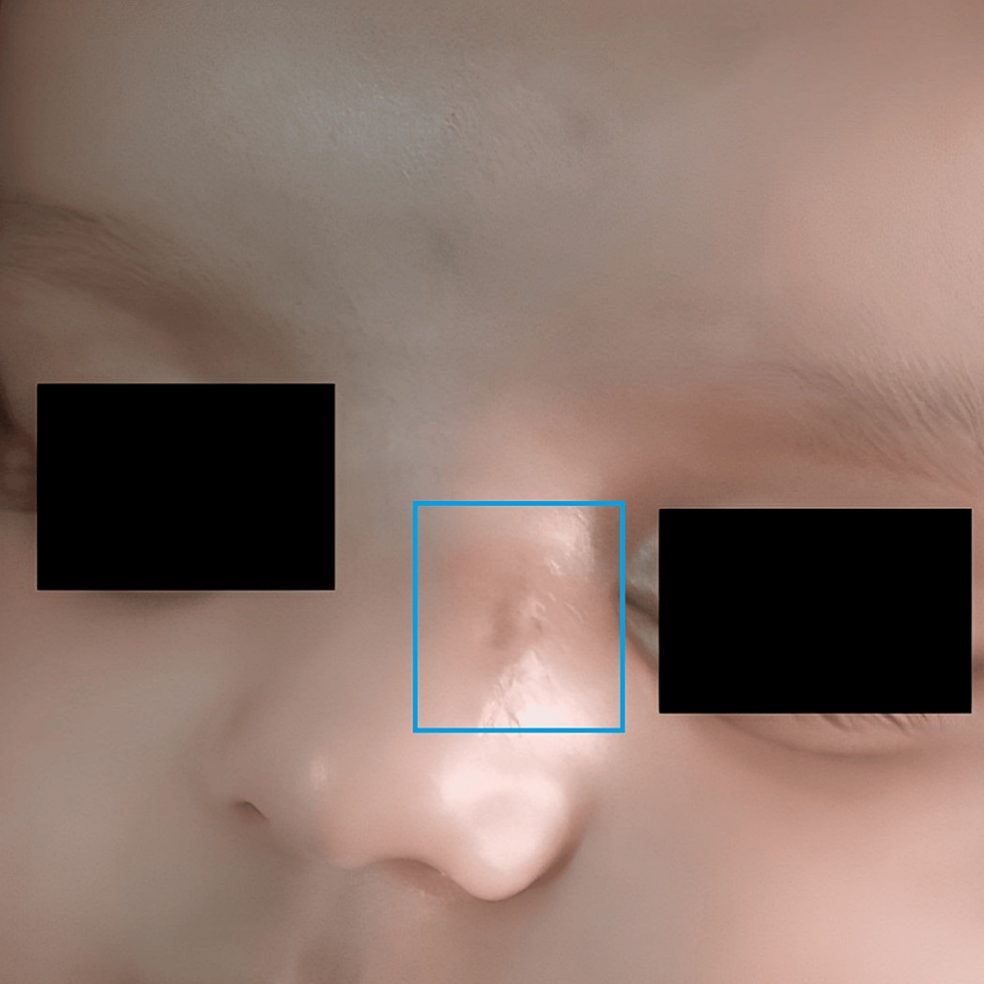
Post-operative site (blue box) of patient.

There were no obvious post-operative complications. The overall cosmetic result was good, notwithstanding the ongoing distortion of the left nasal bone. There was no evidence of a localized lesion recurrence, and the child's condition was satisfactory (Figure 5).

## Discussion

A misleading misnomer, "nasal glioma" suggests a cancerous condition, which is not the case. Nasal gliomas are collections of normal glial tissue in an aberrant position; they must be distinguished from gliomas, which are malignant brain tumours. The presence of mature glial tissue outside the cranial cavity is known as NGH. Some authors have also referred to it as a nasal glioma. Most often, it is identified in early childhood or is present from birth [9].

Ninety percent of the cases are diagnosed before the age of two years. The lesion in this instance has been known about since birth. NGHs can be found inside or close to the nasal cavity; extranasal locations account for 60% of cases, intranasal locations for 30%, and mixed-type instances, which include both extranasal and intranasal locations, for 10% of cases [10]. Firm, smooth masses that do not pulse or swell when you cry, cough, or strain are called extranasal glial heterotopias [9]. They might be linked to hypertelorism. NGH grows relatively slowly and does not have the potential to be malignant, but in cases when it is left untreated, problems including infections or deformities of the septum or nasal bone have been reported. NGH could be an indication of an encephalocele that has severed its intracranial connection. A thin stalk of fibrous tissue attached to the dura as a remnant may be present in 15%-20% of instances. Compared to extranasal variants, intranasal NGH is more likely to have this relationship. In our case, during surgery, no such connection was discovered [11].

NGHs can present as midline nasal masses, but they should be distinguished from encephaloceles and dermoid cysts. Encephaloceles are extracranial hernias of the meninges and/or brain resulting from birth abnormalities related to the cranium. With crying or straining, there is pulsation and an expansion of the mass due to their intracranial connection [6]. It is improbable that our patient has encephaloceles because the mass did not exhibit pulsation and expansion when the patient cried or strained, nor did it reveal any developmental abnormalities or intracranial connections on NCCT. Another differential diagnosis for NGH is a nasal dermoid cyst. A histopathological distinction can be made because the NGH displays mature glial tissue and the skin tissues or dermal appendages such as hair follicles, sebaceous glands, and sweat glands are lined by keratinized stratified squamous epithelium [9]. The choroid plexus, ependymal clefts, and retinal pigmented epithelium are among the rare NGH components that have been documented [11].

Midline swellings in the face can often cause confusion, and since there is a chance of CSF leakage, biopsy should not be used as a standard method of diagnosis. Surgical excision is the treatment of choice [12]. The NCCT in our instance revealed no meningeal or brain communication. Hence, surgical excision was performed and the specimen was sent for histopathology. On histopathological examination, the diagnosis of NGH was confirmed.

## Conclusions

An infant's nasofrontal mass is an uncommon malformation that can lead to serious intracranial problems. NGH should be considered as a differential diagnosis for nasal tumours in newborns and early children, despite its uncommon occurrence. Before performing invasive surgery, proper cross-sectional imaging of the brain, such as CT or MRI, is required to rule out any intracranial connection in cases where nasal gliomas or encephaloceles are suspected. To achieve a definitive diagnosis, a systematic approach including clinical, sonological, and CT or MRI examination should be used; nevertheless, surgical excision and confirmation by histopathological examination are the gold standard. The significance of histopathology in resolving diagnostic conundrums led us to document this rare case.
